# International Hahnemannian Association

**Published:** 1888-02

**Authors:** 


### THE INTERNATIONAL HAHNEMANNIAN ASSOCIATION.

The ninth annual meeting of the International Hahnemannian Association will convene at Niagara Falls in June. The past meetings of this Association have been so interesting and so useful that we must labor diligently during the next six months if we would not have the Niagara meeting fall far behind its predecessors in interest and profit. Therefore let each member regard himself, or herself, as especially called upon to present at that meeting some choice piece of work. Do not leave this work to your neighbor, but be up and doing yourself.

In order to facilitate this work we present here a full list of officers, chairmen, and bureaux, with addresses to render communication easy and certain.

### Officers, 1888.

*President:* William P. Wesselhœft, M. D., 176 Commonwealth Avenue, Boston.

*Vice-President:* Clarence Willard Butler, M. D., Montclair, New Jersey.

*Secretary:* E. A. Ballard, M. D., 97 Thirty-Seventh Street, Chicago.

*Treasurer:* William A. Hawley, M. D., 52 Warren Street, Syracuse, N. Y.

*Censors:* Drs. W. S. Gee, E. Rushmore, C. W. Butler, J. B. Bell, and Dr. Jas. A. Biegler, *chairman*, 58 South Clinton Street, Rochester, N. Y.

*Publication Committee:* Drs. W. P. Wesselhœft, C. W. Butler, and E. A. Ballard.

### Bureaux.

*Philosophy of Homœopathy:* Dr. Ad. Lippe, 1204 Walnut Street, Philadelphia, *chairman*. Members, Drs. P. P. Wells, J. T. Kent, Alice B. Campbell, W. S. Gee, Edward Cranch, Edmund J. Lee.

*Materia Medica, etc.:* Dr. W. S. Gee, Hyde Park, Illinois, *chairman.* Members, Drs. P. P. Wells, Ad. Lippe, J. T. Kent, C. W. Butler, H. C. Allen, H. P. Holmes, E. Rushmore, E. W. Sawyer, H. Hitchcock, C. H. Lawton, E. B. Nash, W. J. Guernsey, Geo. H. Clark.

*Clinical Medicine:* Dr. Alice B. Campbell, *chairwoman.* Members, Drs. E. A. Ballard, J. A. Biegler, C. W. Butler, A. B. Carr, G. H. Clark, W. A. Hawley, H. P. Holmes, W. M. James, J. T. Kent, E. J. Lee, Ad. Lippe, C. F. Millspaugh, E. B. Nash, E. Rushmore, E. W. Sawyer, J. Schmitt, and W. P. Wesselhœft.

*Surgery:* Dr. J. B. Bell, 178 Commonwealth Avenue, Boston, *chairman.* Members, Drs. Edmund Carleton, Thos. M. Dillingham, Julius Schmitt, G. G. Gale. (Name of English physician to be announced later.)

*Obstetrics, Diseases of Women and Children;* Dr. E. P. Hussey, 493 Porter Avenue, Buffalo, N. Y., *chairman.* Members, Drs. W. H. Baker, B. L. B. Baylies, E. A. Ballard, Allen B. Carr, Chas. E. Chase, Samuel L. Eaton, W. J. Hunter Emory, J. B. Gregg Custis, Wm. Jefferson Guernsey, Edward Mahoney, D. C. McLaren, Samuel Long, Franklin Powel, Edward Rushmore, Thomas Skinner, C. Carleton Smith, Julius Schmitt, Rufus L. Thurston, Flora A. Waddell.

